# Supplementary material for: Pan-genome and phylogeny of Bacillus cereus sensu lato
Source: BMC Evol Biol. 2017 Aug 2;17:176. doi: 10.1186/s12862-017-1020-1 (PMC5541404; doi:10.1186/s12862-017-1020-1)
Supplement: Supplementary file 5 — Construction of a HaMStR database. Prokka was used to annotate 114 B. cereus s. l. complete genomes. The resulting protein-coding gene annotations were provided as input to Roary, which constructed a pan-genome consisting of 59,989 orthologous protein sequence clusters. After filtering, which included mobile genetic element removal, the 8954 remaining clusters were aligned with MAFFT. Gene models were built from the multiple sequence alignments using the hmmbuild program from HMMER. The 8954 gene models, together with separately constructed reference taxon BLAST databases, constituted the hamstr_full_mges_removed HaMStR database. (PDF 15 kb) [file 12862_2017_1020_MOESM5_ESM.pdf]

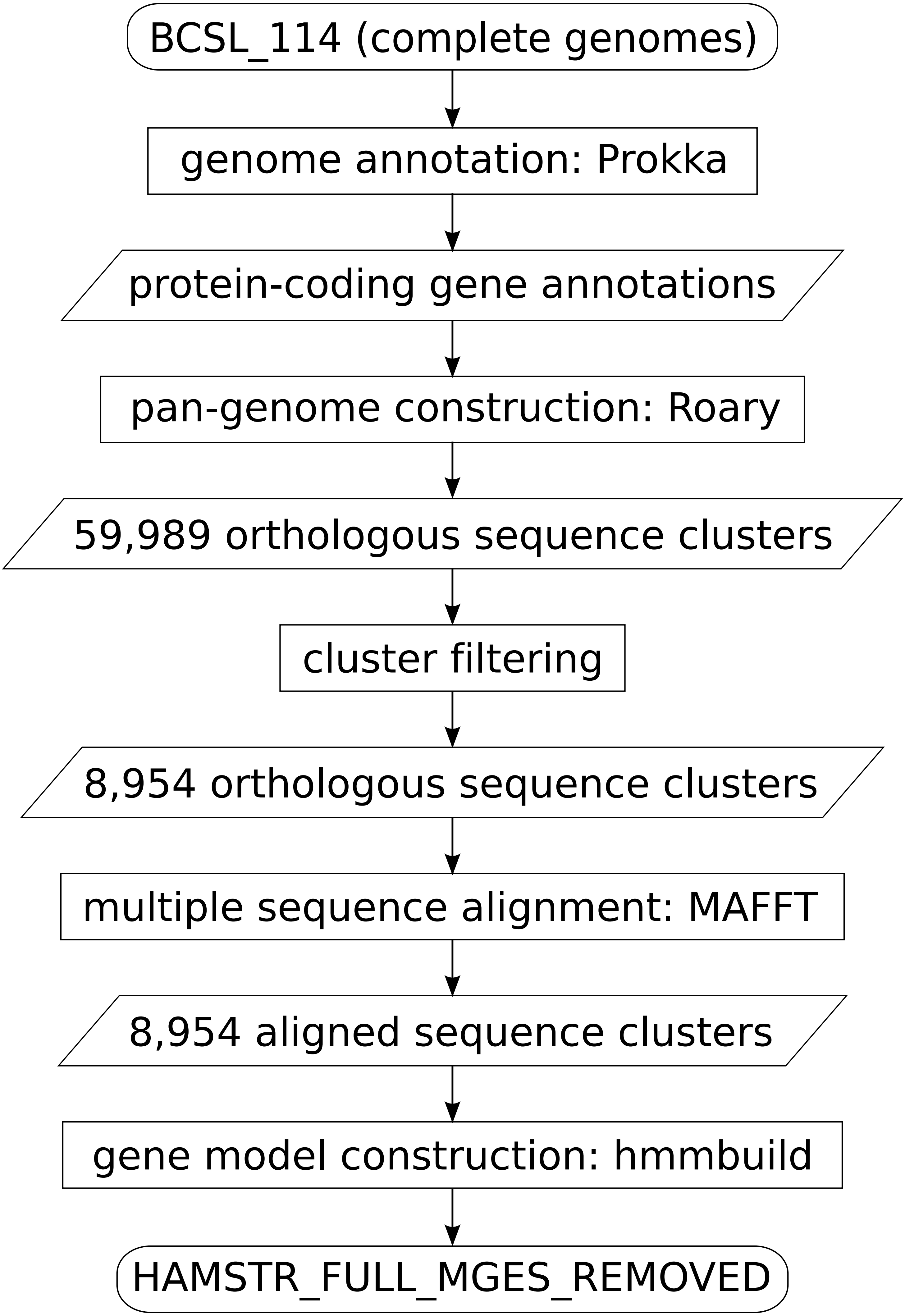
BCSL_114 (complete genomes)
genome annotation: Prokka
protein-coding gene annotations
pan-genome construction: Roary
59,989 orthologous sequence clusters
cluster filtering
8,954 orthologous sequence clusters
multiple sequence alignment: MAFFT
8,954 aligned sequence clusters
gene model construction: hmmbuild
HAMSTR_FULL_MGES_REMOVED
