# Supplementary material for: Pan-genome and phylogeny of Bacillus cereus sensu lato
Source: BMC Evol Biol. 2017 Aug 2;17:176. doi: 10.1186/s12862-017-1020-1 (PMC5541404; doi:10.1186/s12862-017-1020-1)
Supplement: Supplementary file 6 — Construction of a concatenated data matrix. Prokka was used to annotate B. cereus s. l. “query genomes”— i.e., draft genomes that were not included in bcsl_114. The resulting protein-coding gene annotations were provided as input to HaMStR, which used the hmmsearch program from HMMER followed by BLASTP to assign query sequences to HaMStR database gene models. Clusters of orthologous protein sequences from query and database taxa were aligned with MAFFT and converted to corresponding nucleotide alignments. The multiple sequence alignments were reduced to a single sequence per taxon with a consensus procedure that used nucleotide ambiguity codes to combine information from sequence variants where necessary. The individual alignments were then concatenated to produce the final data matrix. (PDF 15 kb) [file 12862_2017_1020_MOESM6_ESM.pdf]

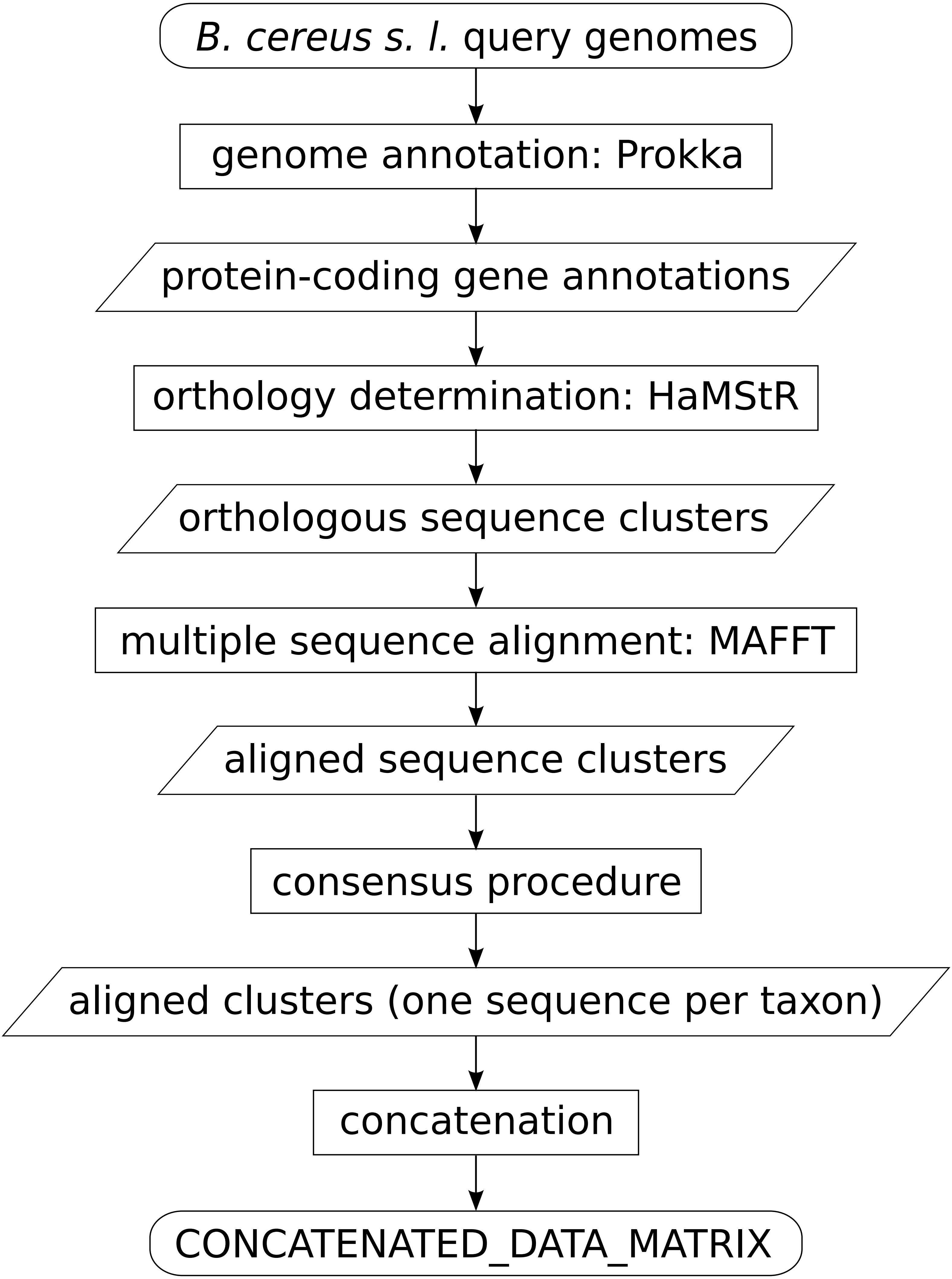
B. cereus s. l. query genomes
genome annotation: Prokka
protein-coding gene annotations
orthology determination: HaMStR
orthologous sequence clusters
multiple sequence alignment: MAFFT
aligned sequence clusters
consensus procedure
aligned clusters (one sequence per taxon)
concatenation
CONCATENATED_DATA_MATRIX
