# Supplementary figures and images for: Pan-genome and phylogeny of Bacillus cereus sensu lato
Source: BMC Evol Biol. 2017 Aug 2;17:176. doi: 10.1186/s12862-017-1020-1 (PMC5541404; doi:10.1186/s12862-017-1020-1)

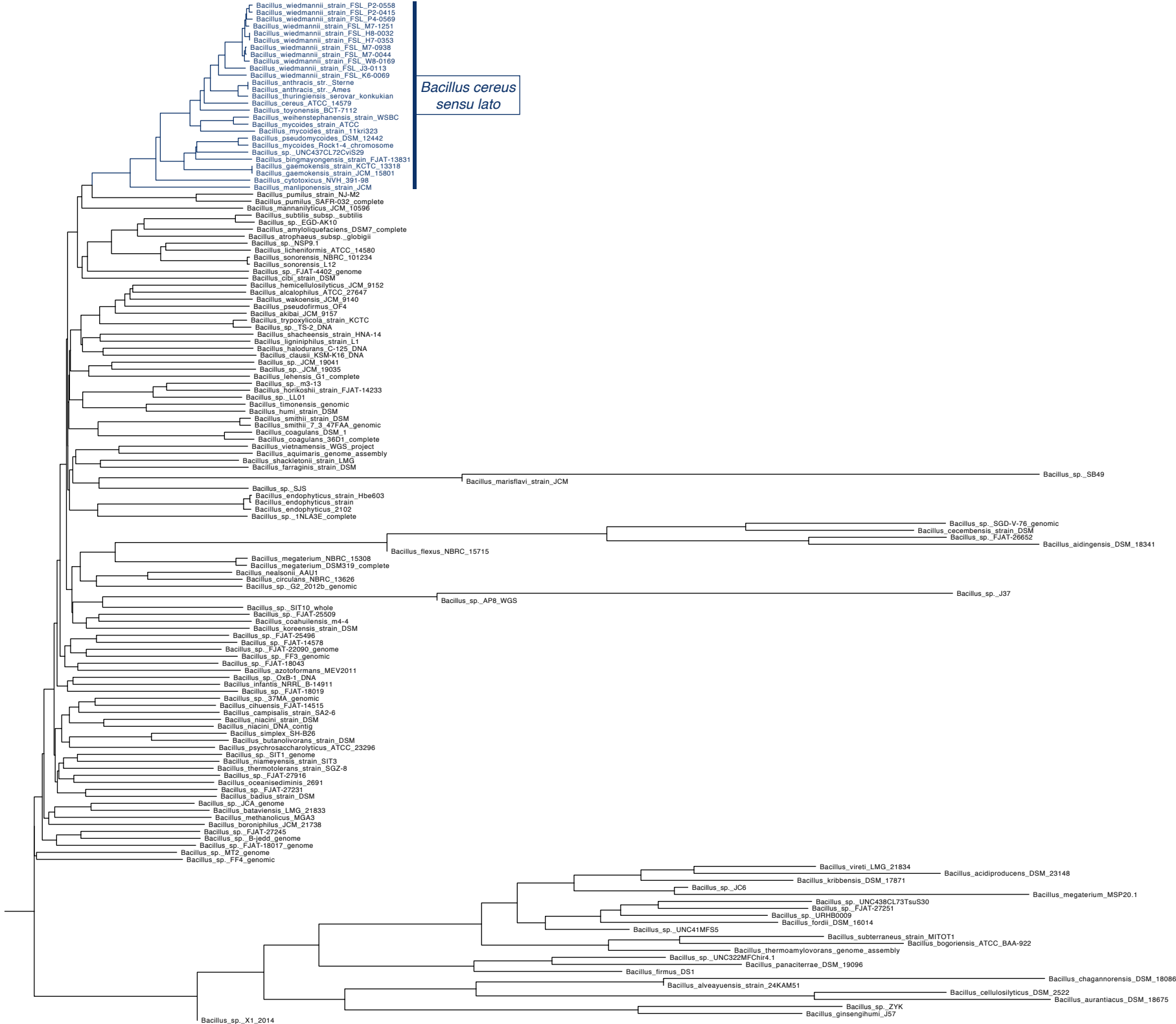

Supplement: Supplementary file 7 — Mash-distance-based phylogeny of the genus Bacillus. Phylogeny of 146 Bacillus genomes, computed with Mash and FastME. (PDF 34 kb) [file 12862_2017_1020_MOESM7_ESM.pdf]

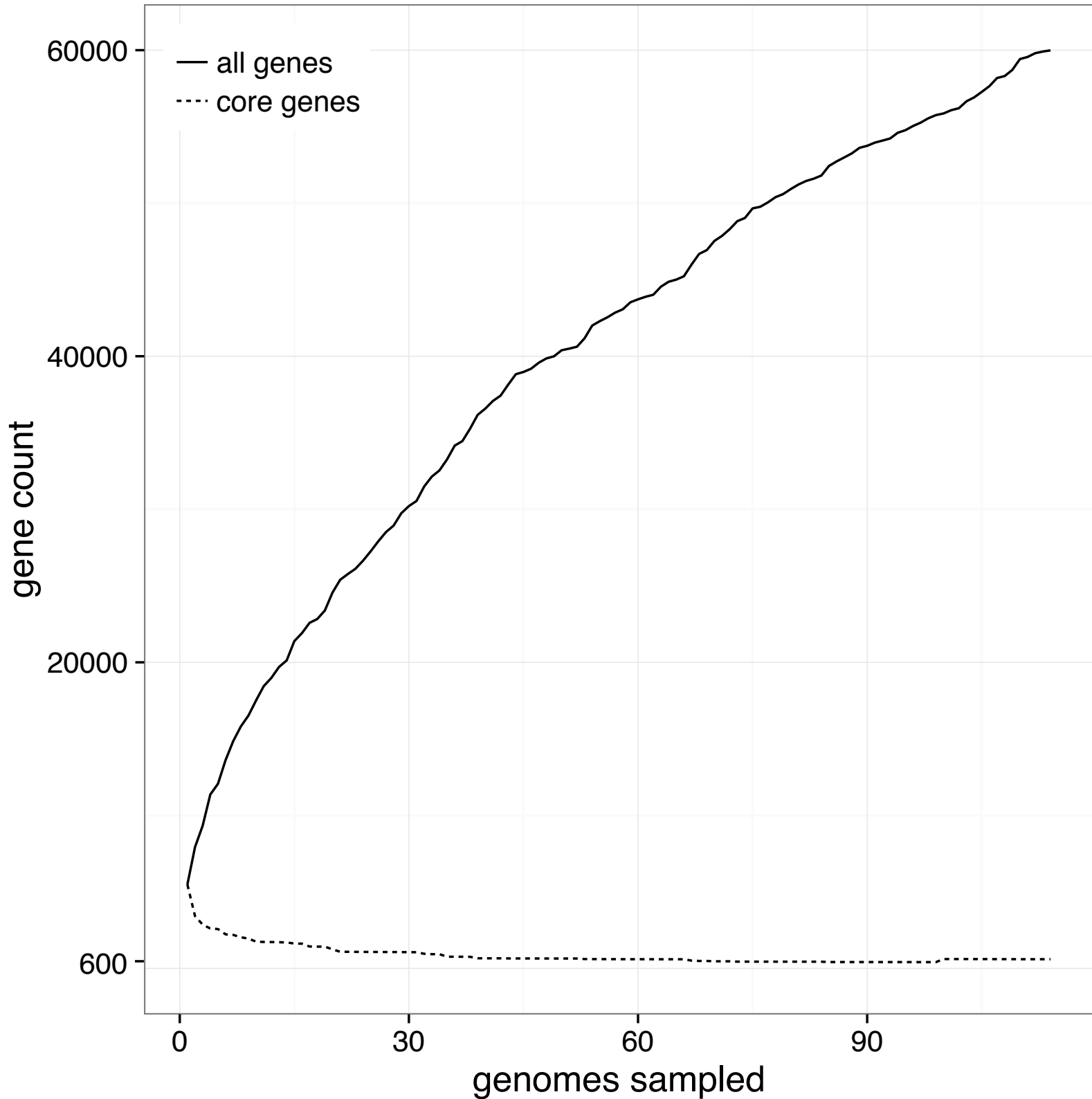

Supplement: Supplementary file 9 — Rarefaction curve: core vs. all genes. The rarefaction curve shows that after ≈35 genomes have been sampled (≈31% of all genomes), the number of core genes remains fairly constant at ≈600 genes, while the total number of genes in the pan-genome continues to increase almost linearly. (PDF 25 kb) [file 12862_2017_1020_MOESM9_ESM.pdf]

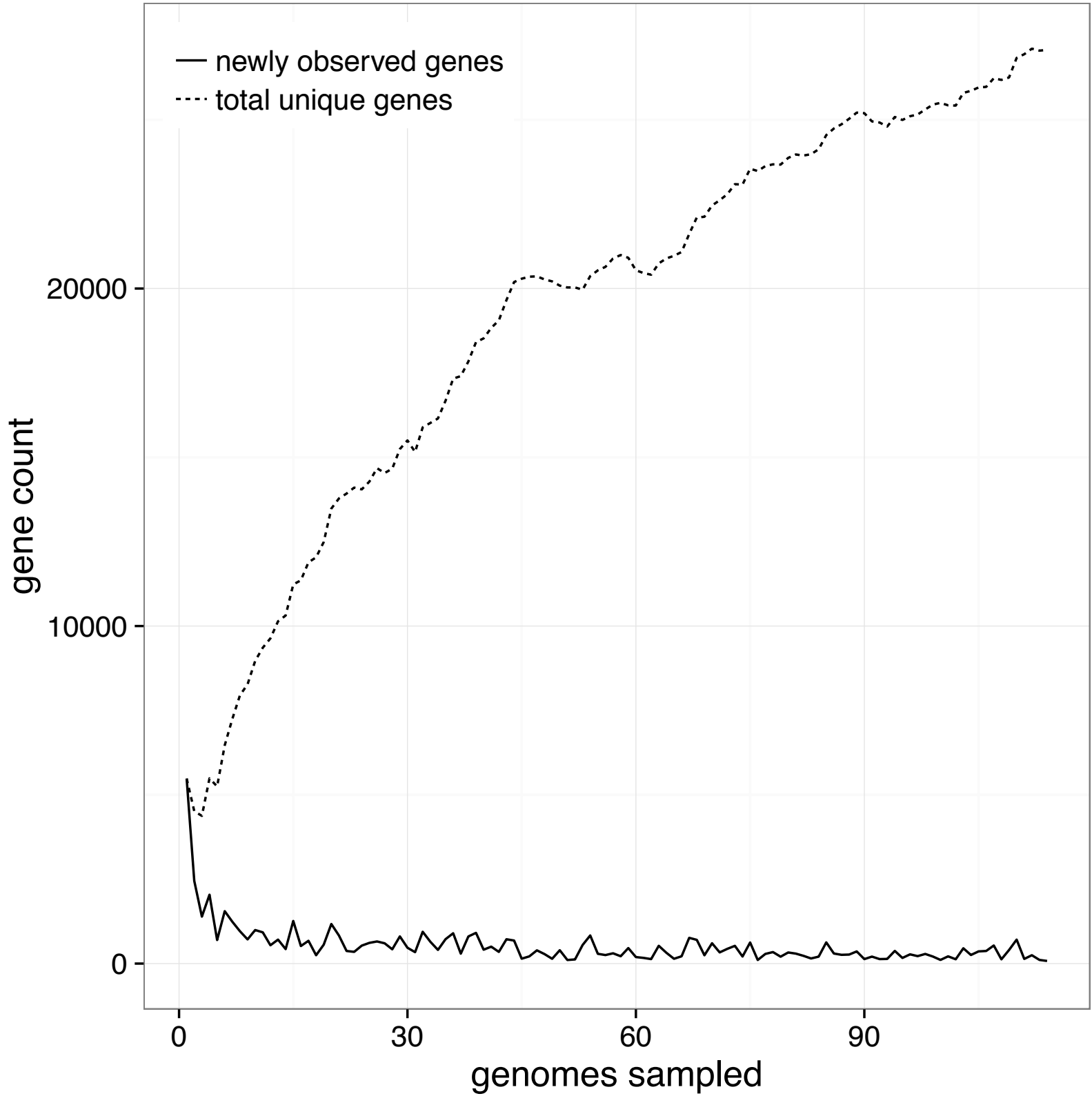

Supplement: Supplementary file 10 — Rarefaction curve: new vs. unique genes. The rarefaction curve shows that as genomes are sampled, genes never before observed continue to be found at a fairly steady rate, and the total number of unique genes discovered continues to increase, with no indication of soon approaching an asymptote. (PDF 15 kb) [file 12862_2017_1020_MOESM10_ESM.pdf]

accessory binary tree  
(114 strains)

gene presence/absence  
matrix (59,989 genes)

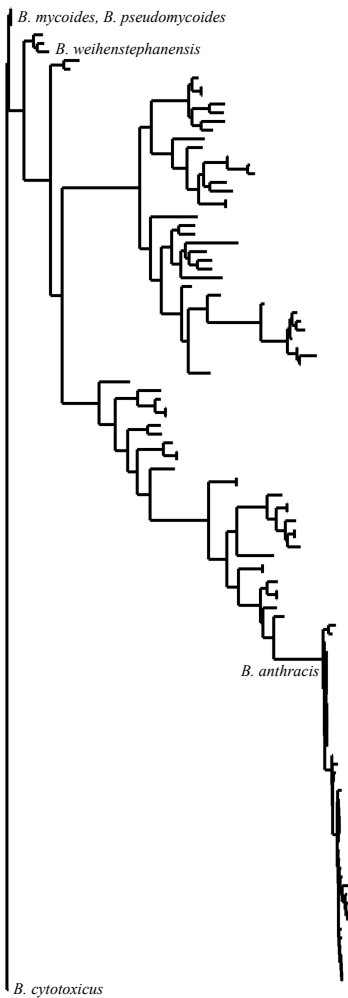

Supplement: Supplementary file 11 — Accessory binary tree and gene presence/absence visualization. The “accessory binary tree” and gene presence/absence information produced by Roary are plotted side-by-side. The outermost B. cereus s. l. clades include taxa with relatively few accessory genes included in the analysis, such as B. cytotoxicus, B. mycoides, and B. pseudomycoides. By contrast, the genomes with the most accessory genes present belong to the highly clonal clade of B. anthracis strains. (PDF 485 kb) [file 12862_2017_1020_MOESM11_ESM.pdf]

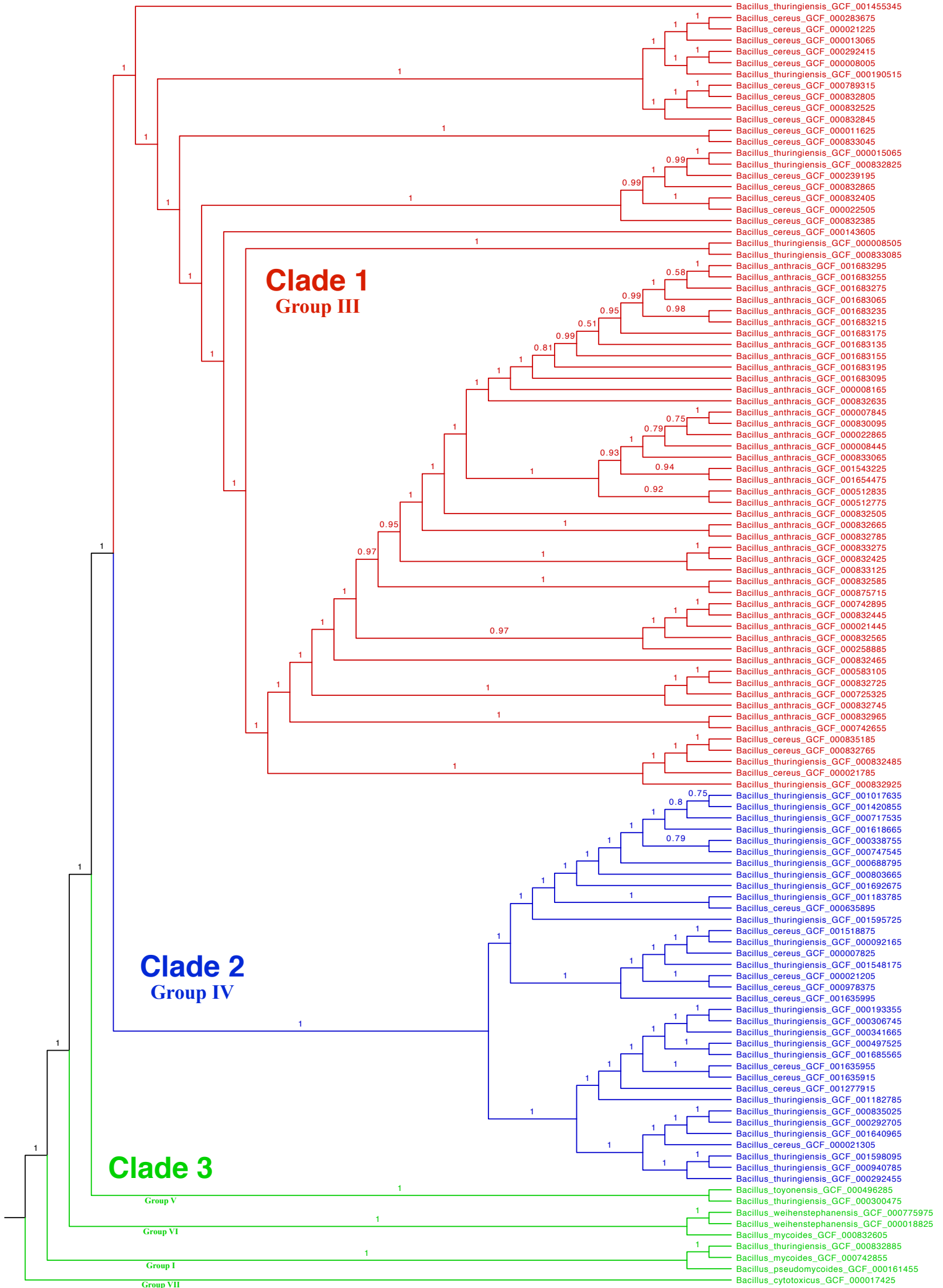

Supplement: Supplementary file 14 — BCSL_114 maximum likelihood phylogenetic analysis results. Cladogram depicting the best estimate of the phylogenetic relationships among bcsl_114 taxa, computed with RAxML using 8954 genes (ml_6; Table 4). B. cytotoxicus was used to root the tree. Major B. cereus s. l. clades and groups are indicated, as are bootstrap probabilities. (PDF 40 kb) [file 12862_2017_1020_MOESM14_ESM.pdf]

**Clade 1**  
**Clade 2**  
**Clade 3**

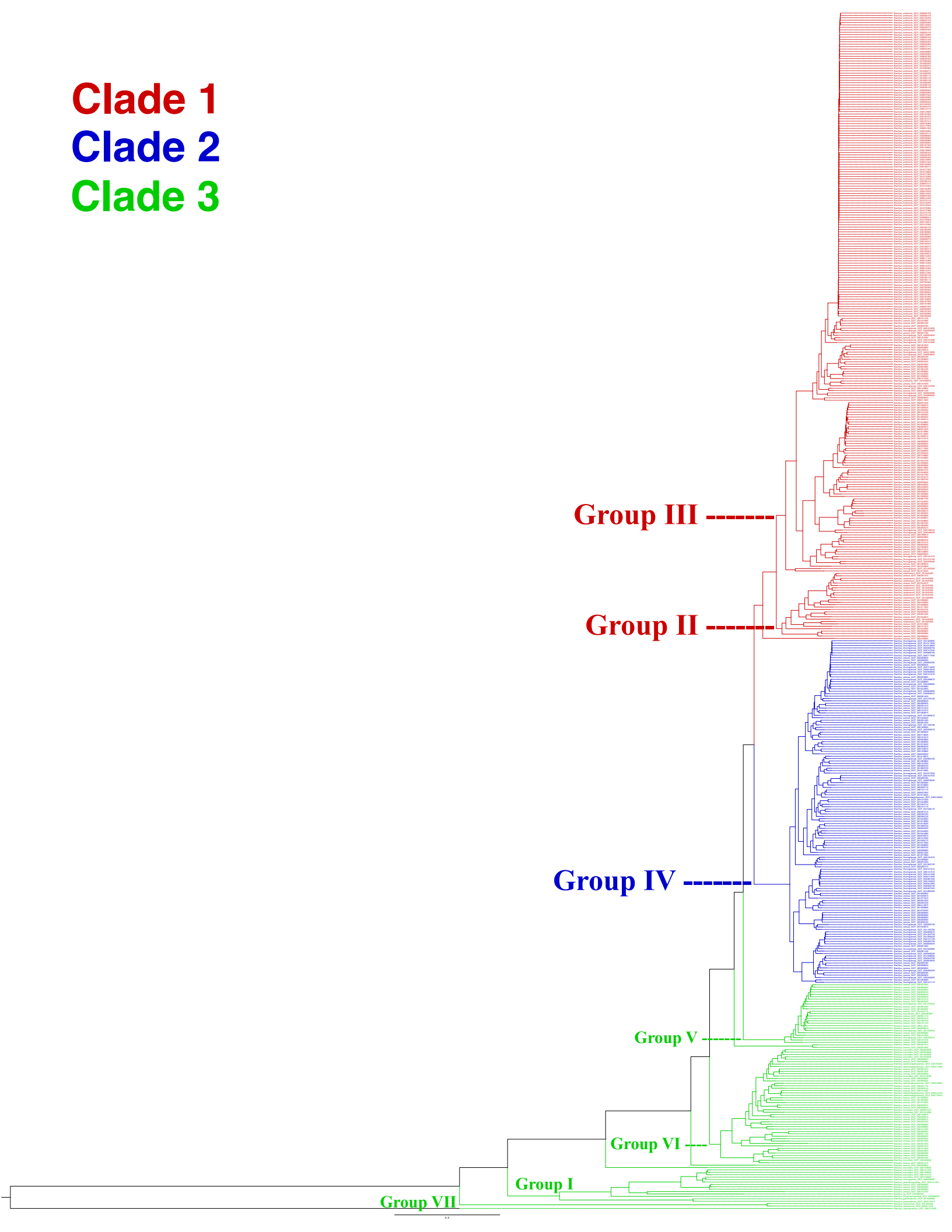

Supplement: Supplementary file 19 — High resolution image of Figure 3. (PDF 76 kb) [file 12862_2017_1020_MOESM19_ESM.pdf]
