# Supplementary material for: Pan-genome and phylogeny of Bacillus cereus sensu lato
Source: BMC Evol Biol. 2017 Aug 2;17:176. doi: 10.1186/s12862-017-1020-1 (PMC5541404; doi:10.1186/s12862-017-1020-1)
Supplement: Supplementary file 16 — BCSL_498 maximum likelihood phylogenetic analysis results, color-coded by species. Phylogram depicting an estimate of the phylogenetic relationships among bcsl_498 taxa, computed with RAxML using 8954 genes (ml_8; Table 4). B. manliponensis was used to root the tree. B. cereus s. l. species tested for monophyly with the gsi are color-coded. (PDF 71 kb) [file 12862_2017_1020_MOESM16_ESM.pdf]

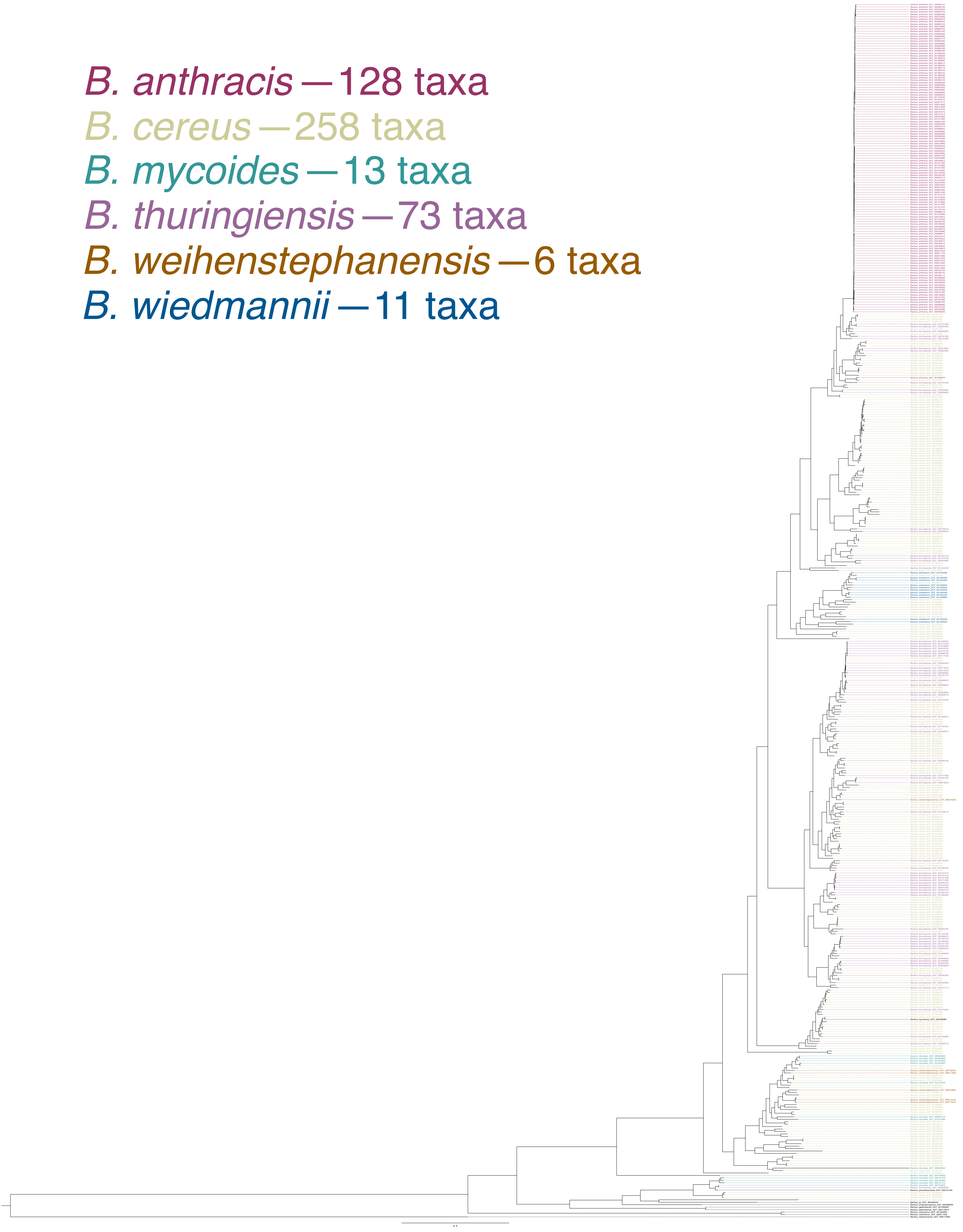
B. anthracis—128 taxa
B. cereus—258 taxa
B. mycoides—13 taxa
B. thuringiensis—73 taxa
B. weihenstephanensis—6 taxa
B. wiedmannii—11 taxa
