# Supplementary material for: Pan-genome and phylogeny of Bacillus cereus sensu lato
Source: BMC Evol Biol. 2017 Aug 2;17:176. doi: 10.1186/s12862-017-1020-1 (PMC5541404; doi:10.1186/s12862-017-1020-1)
Supplement: Supplementary file 17 — High resolution image of Figure 1. (PDF 230 kb) [file 12862_2017_1020_MOESM17_ESM.pdf]

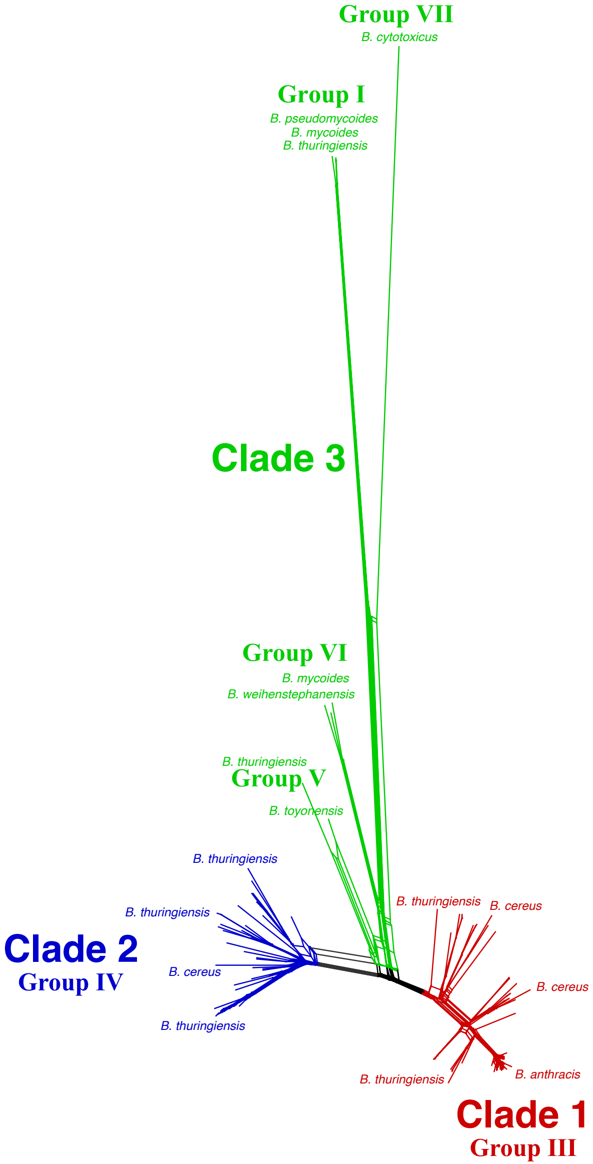
Group VII
B. cytotoxicus
Group I
B. pseudomycoides
B. mycoides
B. thuringiensis
Clade 3
Group VI
B. mycoides
B. weihenstephanensis
B. thuringiensis
Group V
B. toyonensis
B. thuringiensis
B. thuringiensis
Clade 2
Group IV
B. cereus
B. thuringiensis
B. thuringiensis
B. cereus
B. cereus
B. thuringiensis
B. anthracis
Clade 1
Group III
