# Supplementary material for: Pan-genome and phylogeny of Bacillus cereus sensu lato
Source: BMC Evol Biol. 2017 Aug 2;17:176. doi: 10.1186/s12862-017-1020-1 (PMC5541404; doi:10.1186/s12862-017-1020-1)
Supplement: Supplementary file 18 — High resolution image of Figure 2. (PDF 40 kb) [file 12862_2017_1020_MOESM18_ESM.pdf]

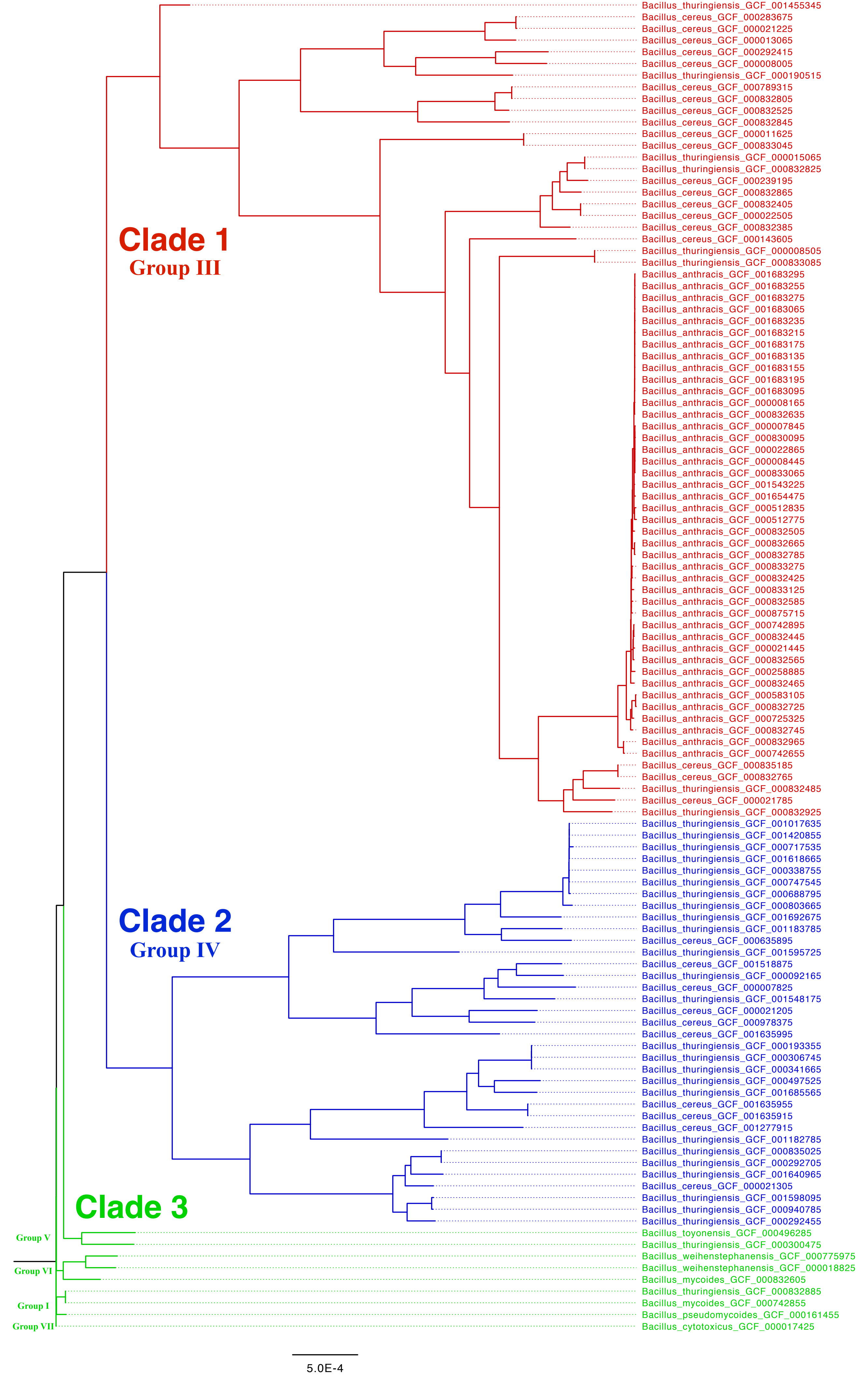
Clade 1
Group III
Clade 2
Group IV
Clade 3
Group V
Group VI
Group I
Group VII
Bacillus_thuringiensis_GCF_001455345
Bacillus_cereus_GCF_000283675
Bacillus_cereus_GCF_000021225
Bacillus_cereus_GCF_000013065
Bacillus_cereus_GCF_000292415
Bacillus_cereus_GCF_000008005
Bacillus_thuringiensis_GCF_000190515
Bacillus_cereus_GCF_000789315
Bacillus_cereus_GCF_000832805
Bacillus_cereus_GCF_000832525
Bacillus_cereus_GCF_000832845
Bacillus_cereus_GCF_000011625
Bacillus_cereus_GCF_000833045
Bacillus_thuringiensis_GCF_000015065
Bacillus_thuringiensis_GCF_000832825
Bacillus_cereus_GCF_000239195
Bacillus_cereus_GCF_000832865
Bacillus_cereus_GCF_000832405
Bacillus_cereus_GCF_000022505
Bacillus_cereus_GCF_000832385
Bacillus_cereus_GCF_000143605
Bacillus_thuringiensis_GCF_000008505
Bacillus_thuringiensis_GCF_000833085
Bacillus_anthracis_GCF_001683295
Bacillus_anthracis_GCF_001683255
Bacillus_anthracis_GCF_001683275
Bacillus_anthracis_GCF_001683065
Bacillus_anthracis_GCF_001683235
Bacillus_anthracis_GCF_001683215
Bacillus_anthracis_GCF_001683175
Bacillus_anthracis_GCF_001683135
Bacillus_anthracis_GCF_001683155
Bacillus_anthracis_GCF_001683195
Bacillus_anthracis_GCF_001683095
Bacillus_anthracis_GCF_000008165
Bacillus_anthracis_GCF_000832635
Bacillus_anthracis_GCF_000007845
Bacillus_anthracis_GCF_000830095
Bacillus_anthracis_GCF_000022865
Bacillus_anthracis_GCF_000008445
Bacillus_anthracis_GCF_000833065
Bacillus_anthracis_GCF_001543225
Bacillus_anthracis_GCF_001654475
Bacillus_anthracis_GCF_000512835
Bacillus_anthracis_GCF_000512775
Bacillus_anthracis_GCF_000832505
Bacillus_anthracis_GCF_000832665
Bacillus_anthracis_GCF_000832785
Bacillus_anthracis_GCF_000833275
Bacillus_anthracis_GCF_000832425
Bacillus_anthracis_GCF_000833125
Bacillus_anthracis_GCF_000832585
Bacillus_anthracis_GCF_000875715
Bacillus_anthracis_GCF_000742895
Bacillus_anthracis_GCF_000832445
Bacillus_anthracis_GCF_000021445
Bacillus_anthracis_GCF_000832565
Bacillus_anthracis_GCF_000258885
Bacillus_anthracis_GCF_000832465
Bacillus_anthracis_GCF_000583105
Bacillus_anthracis_GCF_000832725
Bacillus_anthracis_GCF_000725325
Bacillus_anthracis_GCF_000832745
Bacillus_anthracis_GCF_000832965
Bacillus_anthracis_GCF_000742655
Bacillus_cereus_GCF_000835185
Bacillus_cereus_GCF_000832765
Bacillus_thuringiensis_GCF_000832485
Bacillus_cereus_GCF_000021785
Bacillus_thuringiensis_GCF_000832925
Bacillus_thuringiensis_GCF_001017635
Bacillus_thuringiensis_GCF_001420855
Bacillus_thuringiensis_GCF_000717535
Bacillus_thuringiensis_GCF_001618665
Bacillus_thuringiensis_GCF_000338755
Bacillus_thuringiensis_GCF_000747545
Bacillus_thuringiensis_GCF_000688795
Bacillus_thuringiensis_GCF_000803665
Bacillus_thuringiensis_GCF_001692675
Bacillus_thuringiensis_GCF_001183785
Bacillus_cereus_GCF_000635895
Bacillus_thuringiensis_GCF_001595725
Bacillus_cereus_GCF_001518875
Bacillus_thuringiensis_GCF_000092165
Bacillus_cereus_GCF_000007825
Bacillus_thuringiensis_GCF_001548175
Bacillus_cereus_GCF_000021205
Bacillus_cereus_GCF_000978375
Bacillus_cereus_GCF_001635995
Bacillus_thuringiensis_GCF_000193355
Bacillus_thuringiensis_GCF_000306745
Bacillus_thuringiensis_GCF_000341665
Bacillus_thuringiensis_GCF_000497525
Bacillus_thuringiensis_GCF_001685565
Bacillus_cereus_GCF_001635955
Bacillus_cereus_GCF_001635915
Bacillus_cereus_GCF_001277915
Bacillus_thuringiensis_GCF_001182785
Bacillus_thuringiensis_GCF_000835025
Bacillus_thuringiensis_GCF_000292705
Bacillus_thuringiensis_GCF_001640965
Bacillus_cereus_GCF_000021305
Bacillus_thuringiensis_GCF_001598095
Bacillus_thuringiensis_GCF_000940785
Bacillus_thuringiensis_GCF_000292455
Bacillus_toyonensis_GCF_000496285
Bacillus_thuringiensis_GCF_000300475
Bacillus_weihenstephanensis_GCF_000775975
Bacillus_weihenstephanensis_GCF_000018825
Bacillus_mycoides_GCF_000832605
Bacillus_thuringiensis_GCF_000832885
Bacillus_mycoides_GCF_000742855
Bacillus_pseudomycoides_GCF_000161455
Bacillus_cytotoxicus_GCF_000017425
5.0E-4
