# Supplementary material for: Pan-genome and phylogeny of Bacillus cereus sensu lato
Source: BMC Evol Biol. 2017 Aug 2;17:176. doi: 10.1186/s12862-017-1020-1 (PMC5541404; doi:10.1186/s12862-017-1020-1)
Supplement: Supplementary file 20 — High resolution image of Figure 4. (PDF 164 kb) [file 12862_2017_1020_MOESM20_ESM.pdf]

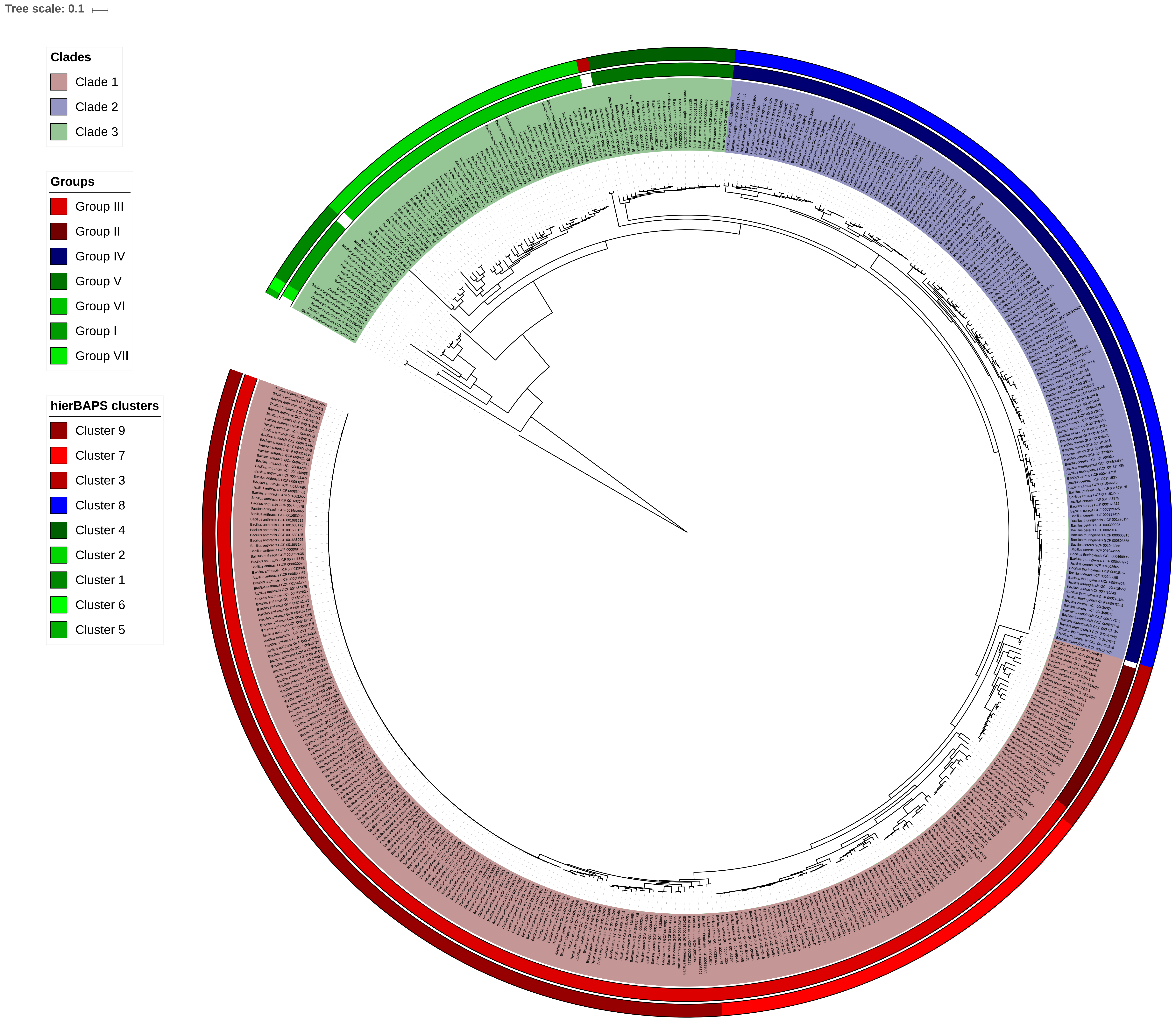
Tree scale: 0.1
Clades
Clade 1
Clade 2
Clade 3
Groups
Group III
Group II
Group IV
Group V
Group VI
Group I
Group VII
hierBAPS clusters
Cluster 9
Cluster 7
Cluster 3
Cluster 8
Cluster 4
Cluster 2
Cluster 1
Cluster 6
Cluster 5
